# Differences in Home Health Nursing Care for Patients with Parkinson's Disease by Stage of Progress: Patients in Hoehn and Yahr Stages III, IV, and V

**DOI:** 10.1155/2021/8834998

**Published:** 2021-02-22

**Authors:** Yumi Iwasa, Izumi Saito, Miyoko Suzuki

**Affiliations:** ^1^Department of Nursing, Graduate School of Health Sciences, Kobe University, Tomogaoka, Suma-ku, Kobe, Hyogo 654-0142, Japan; ^2^Akebi Home-Visit Nursing Station, Hosoe, Shikama-ku, Himeji, Hyogo 672-8064, Japan

## Abstract

As societal aging progresses globally, the number of people with Parkinson's disease (PD) is expected to increase worldwide. Accordingly, the need for home health nursing care for homebound patients with PD will continue to expand. We aimed to clarify the clinical care provided by nurses to homebound patients in each Hoehn and Yahr (HY) stage of the disease. We analyzed the visiting nursing records of patients and observed the clinical care provided by nurses in patients' homes and nursing homes to compare the attributes of patients and differences in nursing care by HY stage. All 21 patients surveyed were at or above HY stage III. The nurses visited each patient nine times per month, on average. The number of visits was positively correlated with HY stage. All stage III patients were homebound, and medication dosage and dispensation assistance were quite common. Several stage IV patients were admitted into nursing homes. In stage V, assistance with hygiene, bedsore management, urine withdrawal/bladder catheters, and other excretory aids were among the most common forms of nursing care provided. As patients' stages progressed, guidance/educational care meant to encourage self-care decreased and direct physical care increased. Clear differences in nursing care were observed between HY stages, suggesting that stage-based protocols regarding the nature and frequency of nursing visits may be useful for ensuring consistent, effective care of patients with PD.

## 1. Introduction

Parkinson's disease (PD) is a neurodegenerative disease with no radical cure. In Japan, patients above stage III on the Hoehn and Yahr (HY) scale are eligible for public medical expense subsidies. When calculated using the Japanese population, the adjusted prevalence of the disease is 166.8 per 100,000 individuals [1].

It is important for patients with PD in long-term medical treatment to receive physical therapy to maintain bodily functions and pharmacotherapy, centering primarily on L-Dopa, to achieve symptomatic relief; both of these efforts require effective care protocols. When provided by nurses, programs that involve counseling, medication information, and collaboration with local organizations during hospitalization and discharge are known to be effective for supporting patients with PD. In the West, PD nurse specialists perform these roles [2–4] and, in recent years, efforts to improve these nursing activities have resulted in studies and surveys leading to the creation of nursing practice guidelines [5–7]. Due to the need to enhance the effectiveness of nursing care provided to patients with PD, the movement to maintain and further improve the quality of care is increasing globally.

Alongside societal aging and economic development, the number of patients with PD is expected to increase globally. Similarly, Japan is also anticipating an increase in the number of patients with PD in the next 20 years. However, there are still no official qualifications for PD nurses in Japan, making this a current topic of debate [8, 9], and there is very little data on the care provided to patients with PD in Japanese hospitals. Furthermore, statistics reveal that the number of patients with PD who use visiting nursing services is large enough to make them the 4th most common type of visiting nursing clients [10]. Dorsey explains how, in the future, nursing for patients with PD will transition from a hospital model to a home-based care model, as the latter can better meet patients' needs at less expense [11]. In Japan, comprehensive regional care systems have been launched to allow patients to reside in familiar environments for as long as possible; thus, it is likely that home health nursing will become even more central for patients with PD in the future. In spite of this need for PD home health nursing, there is a serious lack of data to support this growing field.

For example, there are concerns about both the general lack of uniformity in the current delivery of patient care to people with PD and about home health nursing, generally [7]. In particular, it remains unclear what sort of nursing care should be provided to patients with PD at each stage of the disease. Meanwhile, researchers such as MacMahon report at least three distinct stages regarding patient care needs: in the early stage, giving information; in the middle stages, maintenance therapy; and in the late stages, palliative care. Nevertheless, at the time of this study, there was still little information in the way of uniform procedures and standardized practices for patients in varying stages of PD [12]. Also, the physical capabilities and living situations of patients with PD vary drastically across the stages of the illness. Keus holds that the specific exercise therapies most appropriate for patients vary according to the stage of their disease, and we believe that the same can be said of necessary nursing care [13, 14].

Thus, in this study, we aimed to analyze nursing records and nursing practice observation records to determine what kind of care is currently being provided to patients with PD in their own homes and nursing homes (outside of hospitals). We also sought to understand how patients' care needs vary according to the stages of the disease.

## 2. Methods

### 2.1. Participants and Collected Data

Between July 2019 and December 2019, we surveyed patients with PD using a visiting nursing agency in a western region of Japan. The agency is unique in that it provides home-visit nursing services only to patients with PD. Although there are no official qualifications for PD nurses in Japan, this agency specializes in PD and, therefore, these nurses may have the most expertise for providing PD care. Our research participants were patients whose “visiting nursing order” records indicated “PD” as their primary illness, as determined by their physicians. Eligible patients who provided their consent to participate were included in the study. We collected both the visiting nursing records of these patients and the researcher-administered observation records of the nursing care provided by visiting nurses to these patients, either at their own home or in a nursing home. The attributes of the nurses that provided visiting care were polled via a questionnaire. Nurses were excluded from participation if they did not have either a regular nursing license or an associate nursing license in Japan.

Participating nurses provided their written consent, explicitly agreeing to allow a researcher to observe and accompany them on their visits, to keep a nursing care observation record, and to fill out questionnaires. Participating patients and their families also provided permission for researchers to accompany nurses on visits, collect their nursing records, and observe and record the nursing care activities. All participating patients were registered in the Public Medical Expenses Subsidy in Japan. In other words, all usage fees for home-visit nursing services that exceeded the specified upper limit were free. The maximum amount of self-pay was 30,000 yen per month (about $285 US), even for high-income patients.

The study activities were carried out after the study design was approved by the Ethics Committee of the Graduate School of Health Sciences, Kobe University (no. 821).

#### 2.1.1. Survey of Nurse Attributes and Experience

We surveyed the participating nurses who provide visiting care to patients with PD. The survey was a printed questionnaire regarding home nursing care and was distributed by a team leader of a nursing agency. The leader retrieved the completed surveys from the participants and provided them to us. The survey asked about their age, gender, type of nursing license obtained, years of experience as a nurse, and years of experience as a visiting nurse. We also asked about the number of patients with PD for whom they had provided care.

#### 2.1.2. Visiting Nursing Records

The records of the patients with PD receiving nursing visits were reviewed to obtain demographic information and relevant medical history, including each patient's age, gender, years with PD, and PD progress; the number of people in their household; their place of residence (own home or nursing home); their history of medical complications and required medical treatments; and their Public Medical Expenses Subsidy Certificate registration status (registered or not). The information was available from the nursing record demographic profiles and “visiting nursing orders,” filled out by physicians. Further, information on monthly nursing visits and whether patients also used out-of-home nursing services (day healthcare service) was collected from “visiting nursing report” data. As a scale of progress, we used the HY scale, which is also used in the registration criteria for Japanese Public Medical Subsides and is popular for physicians to complete in the “visiting nursing order.” The HY stage scale used by the Japanese Ministry of Health, Labour and Welfare for the registration criteria of public medical subsides is the same as the HY stage classification provided by the International Parkinson and Movement Disorder Society (UPDRS: the Unified Parkinson's Disease Rating Scale) [15, 16].

#### 2.1.3. Observation Record of Home Health Nursing Care

Using a researcher-administered model for observing and recording the types of patient care performed by visiting nurses, we had one researcher accompanying all the nurses from the station as they visited the participating patients with PD. To ensure that the research conditions were consistent for all visits, the same researcher went on all the visits, regardless of which nurse was assigned to the patient. A 1:1 format was followed for patient visitation (one researcher observed one nurse for only one visit to each patient). The researcher was tasked with recording the care performed by the nurse. One record was kept for each visit, and we selected normal visit scenarios to observe and record. However, for any single patient who had received multiple types of visits—such as rehabilitation visits and dosage visits—the researcher observed and recorded each visit type. Records were collected without interfering with nursing care, and we avoided collecting records during emergencies and other situations where recording would not have been appropriate.

Prior to starting our observational record collection for analysis, we conducted a trial accompanied observation session and confirmed with nurses both the most appropriate recording method and the care items to be recorded. Nursing care items to be documented were determined after consulting Table A of the Japanese Nursing Association's New Nursing Service Category and considering which items were most likely to be relevant to typical care situations [17]. During a particular recording session, if the actual nursing care activities did not correspond to a predetermined item, they were recorded as temporary items. After the visit had concluded, we consulted the nurses that we accompanied and added new care items as needed, which were then observed in future visits.

Records were taken using the “aTimeLogger” application for Android, developed by BGCI. We contacted the application developers to notify them about our research, obtained their permission to use the application, and confirmed that this usage would not breach patient privacy (the application developer would not receive any of the data collected using the app). The application was set up such that there was a corresponding button for each nursing care item to be recorded. When a particular button was pressed, a timer recording the duration of that care item would start. When the next care item was begun, that corresponding button was pressed, causing the previous timer to stop and a new timer for the current nursing care item to start. Thus, the total time for each visit was recorded and categorized by each relevant nursing care activity performed.

### 2.2. Analytical Methods

#### 2.2.1. Analysis of Nurse Attributes

In order to properly understand the characteristics and experience of the target nurse, the mean values of attributes of the nurses (age, years of experience as a nurse, and number of patients with PD who took care of them) were compared to other nurses in Japan.

#### 2.2.2. Analysis of Patient Attributes

To gain an understanding of the overall picture of patients with PD and their characteristics in each HY stage, we compared the attributes between patients in each HY stage based on the demographic and medical history information listed in their nursing records, as described in the above section. Quantitative comparisons were determined using the Kruskal–Wallis test, and Fisher's exact test was used for nominal scale comparisons.

Finally, to understand the relationship between monthly nursing visits received and other patient attributes, we analyzed the correlation between monthly nursing visits and each of the following using Pearson's correlation coefficient: age, years with PD, and the number of individuals in the household. The correlation between monthly nursing visits and HY stage was analyzed using Spearman's correlation coefficient.

#### 2.2.3. Analysis of Home Health Nursing Care Items

To clarify the specific nursing care items associated with the patients' HY stages, we compared the percentage of patients receiving each item across each stage. A particular care item was considered “received” if it was observed as being performed for any length of time during a visit and was considered “not received” if not observed. In the case of patients who were observed multiple times, a particular care item was considered “received” if it was observed in at least one session. Care reception percentages were compared using Fisher's exact test. All statistical analyses were done in the SPSS Statistics 26 (Windows 10) application (IBM), and the threshold for statistical significance was set at 5%.

## 3. Results

### 3.1. Nurse Overview

Five nurses performed the home health nursing care observed by the researchers. All nurses were licensed as Japanese regular nurses. An overview of their attributes can be seen in Table 1. Their mean age was 54.60 ± 10.15 years and they had, on average, 32.05 ± 8.23 years of nursing experience (Table 1). The average number of patients with PD that each of the participating nurses had visited prior to this study period was 65.00 ± 40.42.

### 3.2. Overview of 1:1 Visits, Patient Attributes, and Comparisons between HY Stages

Visiting Nursing Station A was overseeing home health nursing care for 24 patients with PD at the time of the study; of these, we obtained research consent from 21. Twenty-one patients of the visiting nursing station were visited separately by five nurses at that station (which nurse visits which patient is randomly assigned, based on the schedule). The same researcher observed every visit; there were 24 visits in total. The overview of the attributes of the patient participants can be seen in Table 2. The median age of participants was 78.0 years, and the median number of years with PD was 13.0. Nine patients were male (42.9%), and 12 were female (57.1%). Seven patients were in HY stage III (33.3%), eight were in stage IV (38.1%), and six were in stage V (28.6%). None of the patients were in stage I or II. Twelve patients were living in their own homes (57.1%), and nine lived at a nursing home (42.9%.) The median number of individuals in a patient's household was 2.0. The median number of monthly nursing visits received per patient was nine, 20 (95.6%) patients went out of their homes to receive care from a “day healthcare service,” and 100% of patients were registered for a Public Medical Expenses Subsidy Certificate. Dementia (13 patients, 61.9%) and cardiovascular and orthopedic illnesses (9 patients for each, 42.9%) were the most common comorbidities, and the most common medical treatment was urine withdrawal/indwelling bladder catheters (four patients, 19.0%).

Comparisons between HY stages yielded no significant results for age and gender. The fewest number of individuals was found in the households of patients in HY stage IV, as was the highest percentage of patients living in nursing homes. Both of these parameters exhibited significant interstage differences. A significant difference in the number of monthly nursing visits was also observed between patients at different HY stages, with the number of visits increasing as HY stage increased. In terms of comorbidities, urological illness was significantly more common in individuals at HY stage V, but no significant differences were observed between stages and any other illnesses or conditions. As for medical care, the need for urine withdrawal/indwelling bladder catheters was significantly more common among patients at HY stage V. Both aforementioned significant differences had *p* values less than 0.05. Finally, patients requiring bedsore treatment were most common among those in HY stage V, and this difference tended toward significance (*p* < 0.1). No significant interstage differences were observed for other forms of medical care.

### 3.3. Nursing Visits and Patient Characteristics

Correlations between monthly nursing visits and age, years with PD, HY stage, and the number of people in the patient's household are listed in Table 3. A significant positive correlation was found between visits and HY stage (*p* < 0.05), but only a weak positive correlation was found between visits and years with PD. A weak negative correlation was found between visits and the number of people in a household (*p* < 0.1).

### 3.4. Implementation of Nursing Care Items and Interstage Comparisons

**Figure 1:**
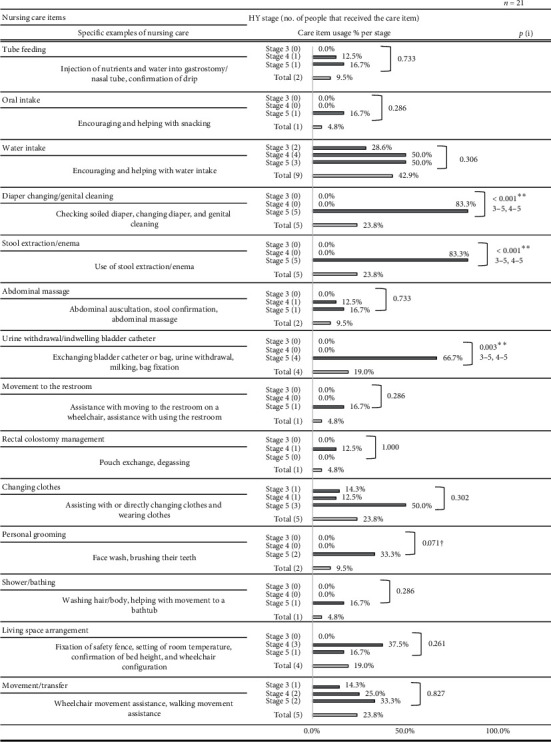
Comparisons of the percentage of nursing care provided at each HY stage. (i) Fisher's exact test (every side) indicates significantly different pairs (p < 0.05) after Bonferroni correction; †p < 0.1, ^∗^p < 0.05, and ^∗∗^p < 0.01; p values and percentages have been rounded off.

**Figure 2:**
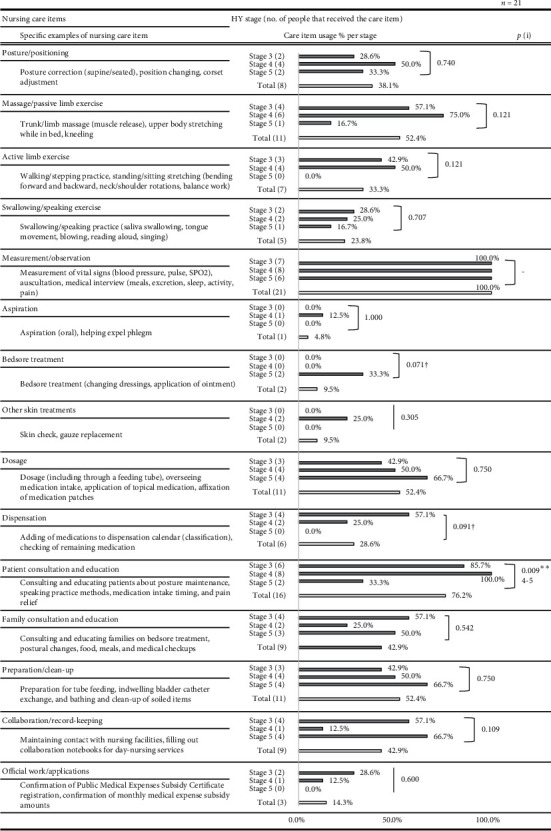
Comparisons of the percentage of nursing care provided at each HY stage. (i) Fisher's exact test (every side) indicates significantly different pairs (*p* < 0.05) after Bonferroni correction; ^†^*p* < 0.1, ^*∗*^*p* < 0.05, and ^*∗∗*^*p* < 0.01; *p* values and percentages have been rounded off.

Over the course of 24 nursing visits, the 21 patients with PD whom we observed received a total of 17 hours, 39 minutes, and 7 seconds of directly observed nursing time. The median length of a single nursing visit was 47 minutes, 2 seconds. After consulting with the participating nurses, a list of 29 care items was created. Figures 1 and 2 list the per-stage breakdown of the percentage of patients in that stage to whom a given care item was performed (for any length of time), as well as specific examples of care practices.

The nursing care items most commonly performed across all HY stages and their implementation rates are as follows: measurement/observation (100%); patient consultation/education (76.2%); dosage, massage/passive limb exercise, and preparation/clean-up (each 52.4%); and water intake, family consultation/education, and collaboration/record-keeping (each 42.9%). Significant interstage differences in implementation rates were found for the following nursing care items: diaper changing/genital cleaning, stool extraction/enema, urine withdrawal/indwelling catheter care, and consultation/education for patients (*p* < 0.05). The interstage differences in the implementation rates of personal grooming, bedsore treatment, and dispensation tended toward significance (*p* < 0.1).

## 4. Discussion

### 4.1. Nurses' Characteristics

On average, the nurses surveyed in this study were 54.60 years old and had 32.05 years of experience; both of these figures were slightly higher than the national averages for all of Japan (47.0 years old and 22.3 years of experience) [18]. Japan does not have a widely employed qualification equivalent to the PD Nurse Specialist designation employed in the UK. Thus, while the nurses in this study did not have such extensive experience as specialized nurses abroad—such as those in the Netherlands, where guidelines propose a requirement of 150 patients treated, or UK, where the criterion is 500—we believe they have enough years of nursing experience to provide appropriate care to patients with PD [6, 7].

### 4.2. Characteristics of Patients with PD and Nursing Care

#### 4.2.1. Characteristics across all Stages

The median number of monthly nursing visits received by the patients studied here was nine, slightly higher than the mean for patients with PD throughout Japan [10]. Monthly visit counts exhibited a strong positive correlation with HY stage, making it clear that patients in advanced stages of PD required more frequent nursing care. These findings suggest the patients studied here tended to be slightly more advanced in their illness, overall.

In addition, nearly all of these patients left their homes to receive care from day healthcare services. Thus, we can surmise that in their daily lives, these patients make use of additional social support besides visiting nursing services.

Of our 29 nursing care items, measurement/observation, patient counseling/education, dosage, and massage/passive limb exercise were the most common. These results demonstrate that the nurses not only encourage self-care and provide psychological support but also work to maintain the functionality of patients' limbs, provide palliative care, and place great importance on medication. However, this study focused on practical nursing care, and we only observed nursing care activities performed by nurses while visiting their patients. For this reason, even if sleep management advice and psychological support, which MacMahon emphasizes as particularly important, were provided by the nurses we observed, these dimensions did not surface as salient elements of care [12]. Finally, caseload management, whose practice guidelines hold to be a critical part of care, was done by the nurses after they returned to their offices, so it was not counted as a practical element of care; this fact should be kept in mind when considering our results or planning future studies [5, 6].

The following characteristics typified the care that we observed being implemented to patients in each HY stage.

### 4.3. Patient and Nursing Care Characteristics by HY Stage

#### 4.3.1. Characteristics of Patients and Nursing Care in HY Stages I and II

In this study, no home health nursing care was provided to patients in HY stage I or II. Besides the fact that advanced patients are in more dire need of nursing care, stage I and II patients, who are less severely ill than patients in other stages, cannot register with the Public Medical Expenses Subsidy system. Thus, even if they require nursing care, it may cause them significant financial burden. Zhao reports that a median time of about two years was found in all HY stage transitions except for the transition between stages II and 2.5, which reportedly takes approximately five years [19]. The nursing care required during the long time spent in stage II is an area for future research to explore. Depending on the frequency of nursing visits and the items of nursing care, it is necessary to consider the need for support for patients in these stages from the Public Medical Expenses Subsidy system.

#### 4.3.2. Characteristics of Patients and Nursing Care in HY Stage III

All patients in stage III were living in their own homes. The median number of people in the patients' household was highest in this stage, at 3.0. We believe that patients at this stage are able to maintain relatively independent lives with the help of their families. The median number of nurse visits received by patients in this stage was 8.0, which corresponds to two visits per week. A weak negative correlation was observed between the number of visits and the number of individuals in the patient's household; this may be driving the low number of visits seen here.

In terms of nursing care, rather than dosing patients directly, nurses more commonly allotted medications for patients in stage III, helping them maintain regular and correct regimens. This practice is common because care that helps patients maintain medication adherence is crucial to prolonging and elevating the effects of L-Dopa formulations. One characteristic of this stage appears to be that care involving observation of patients' blood pressure or general condition and education on rehabilitative care for family members was quite common; however, direct care surrounding meals or hygiene was rather uncommon. Thus, nursing care at this level was typified by indirect, supportive, and educational care meant to assist patients' ability to live independently.

#### 4.3.3. Characteristics of Nursing Care and Patients in HY Stage IV

From stage IV onward, the number of patients living in nursing homes significantly increased. The at-home living environment and patients needing increasingly more lifestyle care make it more difficult for them to live in their own homes, prompting them to make use of residential social support services in their daily lives. In terms of nursing care, care dealing with physical movement also gradually increased. This suggests that the need for physical, direct nursing care becomes more common in stage IV. The median number of monthly nursing visits for patients in this stage was 11.0, corresponding to an average of three visits per week. We believe that the increase in patient counseling/education seen in this stage was driven both by increased living difficulties and the large number of people living alone at this stage compared to stage III.

While no significant differences were observed for massage/passive limb exercise and postural maintenance, they were performed most frequently in stage IV, suggesting that patients in this stage had more nonmotor symptoms, such as joint and muscle pain, prompting nurses to provide palliative care. Care involving patient education and consultation continued to be performed in this stage, but the most salient characteristic of stage IV was an increase in nursing care meant to directly support and alleviate the difficulty in activities of daily living due to declines in functionality.

#### 4.3.4. Characteristics of Patients and Nursing Care in HY Stage V

Some individuals in stage V were still living at home, receiving care from their family members. It is unclear why there were more female patients in this stage. The median number of monthly nursing visits received by patients in this stage was 17.5, which corresponds to approximately four visits per week. Patients at this level spent a lot more time in their beds, and nursing care involving the treatment of bedsores tended to be necessary. Further, patients' physical mobility decreased, and the need for care such as diaper changes and personal grooming increased considerably. Patients were also unable to take medications on their own and tended to need dosage support from nurses.

Additionally, patients with urological complications increased at this level. Nonmotor symptoms grow quite advanced at this stage, meaning that urine withdrawal/indwelling bladder catheter care was much more commonly performed in HY stage 5. We believe that the increase in stool extraction and enema care at this stage was also due to progression of the disease, which causes severe constipation.

The decrease in patient consultation/education seen in stage V is likely due to the fact that as many as 83.3% of the patients in this stage had dementia. However, the rates of family consultation/education increased. According to Shin, support for caregiving family members should be of the utmost priority, especially for nurses attending to patients in advanced stages of disease [20]. While we did not make detailed observations thereof, significant interstage differences in the content of consultation and education provided to both patients and family members existed, and these differences were important.

While these differences did not reach statistical significance, in HY stage V, no patients were undergoing active exercise, and few received massages or passive limb exercise. It may be alarming that palliative care, such as massages or passive limb exercises, is diminishing despite frequent visits at the most advanced stage. However, swallowing/speaking exercises continued to be practiced, indicating that rather than care focused on motor functions of the limbs, care aimed at maintaining basic functions such as eating and speaking is a priority for patient care in stage V. A characteristic of this stage is, therefore, nursing care that directly addresses patients' basic physical needs in addition to tasks assisting excretion and maintaining hygiene. However, nurses may not be able to provide palliative care to those in whom the disease is most advanced, either because physical care is a priority, or because patients cannot request care for themselves due to dementia.

### 4.4. Limitations of This Study

Goetz et al. say the following about HY scale classifications: [the HY scale] “weighted heavily toward postural instability as the primary index of disease severity, it does not capture completely impairments or disability from other motor features of PD and gives no information on nonmotor problems” [21] (p. 22). It is essential to note that motor symptoms apart from postural instability and nonmotor symptoms do not progress according to the pace of PD stage transitions. However, Goetz et al. also state that progressively higher stages correlate with neuroimaging studies of dopaminergic loss, and high correlations exist between the HY scale and some standardized scales of motor impairment, disability, and quality of life [21]. The results of this study also indicate that, as motor symptoms progress, patients' lifestyles change, as does the nursing care they require. In particular, as patients progressed from stage III to stage V, nursing care changed from guidance/educational care meant to encourage patient and family self-care to direct physical care. Of course, while it is not always clear what stage a patient may be in, it is vital for nurses to not only understand the standard nursing care practices for nursing home and at-home patients, the frequency with which they must be performed, and the characteristics thereof, but also to develop the ability to predict how these aspects may change as these patients' diseases progress.

However, the frequency and nature of home health nursing care varied not only with the patients' stage of disease, but also with their living situation and number of individuals in their household. Furthermore, while 1/3 of nurses surveyed by Axelrod et al. were qualified to prescribe medications, in Japan, nurses cannot write prescriptions; that is, differences in the nursing systems of different countries will impact the kind of care nurses therein can perform [6]. Therefore, we must not forget that a myriad of factors apart from patients' HY stage can cause differences in provided care.

This study was a single-agency small-sample study. Although the change in care must be an international trend, similar investigations must be done at other agencies as well to corroborate our results. Additionally, we must carry out follow-up observations to ascertain the nature of and frequency with which counseling/educational care—which is critical to patients with PD—was provided to patients in each stage. Finally, follow-up observations may also reveal what sort of care activities are undertaken by nurses when they are not in their patients' presence. Despite these limitations, this pilot study is valuable because it informs the design of more comprehensive and multicenter observations of nursing activities according to patients' PD stage.

## 5. Conclusions

Records kept by visiting nursing services indicate that the median number of monthly visits required by patients with PD was nine. We found that the number of visits recorded increases as the patient's disease progresses through the stages of the disease. A similar effect was observed for the patients' place of residence (patients' homes or nursing homes), which consistently changed as the disease progressed.

After analyzing the researchers' observation records of nursing care agency visits to patients with PD, we determined the following: in HY stage III, the most common nursing care is provided to help patients regularly take their medications on their own; in stages III and IV, patient consultation/education was most common. In stage V, stool extraction/enema, urine withdrawal/indwelling bladder catheter care, bedsore treatment, diaper changing, and personal grooming care are most common, whereas patient consultation/education is uncommon. Thus, as HY stages increases, the nursing care provided to patients with PD becomes increasingly physically direct. We must understand how nursing care needs can change and endeavor to predict such changes before they occur.

The results of this study may aid the design of standardized protocols for home health nursing care for patients with PD based on the stage of the disease. Anticipating the frequency of visits and the type of support required by patients at each HY stage of PD may allow for more efficient use of the time and resources provided for home health nursing care.

The visiting nurse programs should be restructured, with training provided to the nurses relevant to the specific needs of patients in each stage of progression. To ensure consistent quality of care to all patients in the program, protocols and checklists should be provided for the visiting nurses attending patients with PD, with some flexibility built in to address individual patient needs. Establishing protocols and planning based on disease progression may promote more consistent, high-quality care for patients as they live with the progression of PD.

## Figures and Tables

**Table 1:** Nurse overview.

	*n*
*Overall*	5
(Breakdown)	
Male	0
Female	5
Regular nurse	5
Associate nurse	0

*Attributes, etc.*	Mean ± SD
Age	54.60 ± 10.15
Years of experience as a nurse	32.05 ± 8.23
Years of experience as a visiting nurse※	6.50 ± 3.24
PD patients they have provided nursing care to (no. of people)※	65.00 ± 40.42

※*n* = 4.

**Table 2:** Patient overview and comparisons by HY stage.

	Total	HY stage 3	HY stage 4	HY stage 5		
	*n* = 21	*n* = 7 (33.3%)	*n* = 8 (38.1%)	*n* = 6 (28.6%)		

(Attributes, living situation)	Median	Median	Median	Median	*p* (i)	Stage comparisons
Age	78.0	77.0	78.0	82.0	0.377	
Number of years with PD	13.0	11.0	14.0	13.0	0.050^†^	
Number of people in household (including self)	2.0	3.0	1.0	1.5	0.048^*∗*^	
Monthly uses of visiting nursing service	9.0	8.0	11.0	17.5	0.004^*∗∗*^	3&5

	No. of people (%)	No. of people (%)	No. of people (%)	No. of people (%)	*p* (ii)	
(Individuals to whom the following applies)						
Male	9	4	4	1	0.355	
	42.9%	57.1%	50.0%	16.7%		
Lives in a nursing home	9	0	6	3	0.011^*∗*^	3&4
	42.9%	0.0%	75.0%	50.0%		
Registered for Public Medical Expenses Subsidy Certificate	21	7	8	6	—	
	100.0%	100.0%	100.0%	100.0%		
Uses a day-caring service	20	7	8	5	0.286	
	95.2%	100.0%	100.0%	83.3%		

(Individuals with the following complications)						
Cardiovascular disease (Including high blood pressure)	9	4	3	2	0.742	

	42.9%	57.1%	37.5%	33.3%		
Respiratory disease	3	0	1	2	0.253	
	14.3%	0.0%	12.5%	33.3%		
Digestive disease	4	1	2	1	1.000	
	19.0%	14.3%	25.0%	16.7%		
Orthopedic disease	9	3	3	3	1.000	
	42.9%	42.9%	37.5%	50.0%		
Cerebrovascular disease (besides PD)	3	2	1	0	0.600	
	14.3%	28.6%	12.5%	0.0%		
Urological disease	6	1	1	4	0.073^†^	3&5
	28.6%	14.3%	12.5%	66.7%		4&5
Diabetes	3	2	0	1	0.347	
	14.3%	28.6%	0.0%	16.7%		
Dementia	13	3	5	5	0.391	
	61.9%	42.9%	62.5%	83.3%		

(Individuals requiring medical treatment or management)						
Tube feeding	2	0	1	1	0.773	
	9.5%	0.0%	12.5%	16.7%		
Aspiration (oral)	2	0	1	1	0.733	
	9.5%	0.0%	12.5%	16.7%		
Bedsore care	2	0	0	2	0.071^†^	
	9.5%	0.0%	0.0%	33.3%		
Urine withdrawal/indwelling bladder catheter	4	0	0	4	0.003^*∗∗*^	3&5
	19.0%	0.0%	0.0%	66.7%		4&5
Home oxygen	1	0	0	1	0.286	
	4.8%	0.0%	0.0%	16.7%		
Colostomy	1	0	1	0	1.000	
	4.8%	0.0%	12.5%	0.0%		

Monthly nursing visits were calculated from visiting nursing report data; (i) Kruskal-Wallis tests (Independent Samples); (ii) Fisher's exact test (every side); ^†^*p* < 0.1, ^*∗*^*p* < 0.05, ^*∗∗*^*p* < 0.01. The “Stage comparisons” column indicates the pair of stages that were determined to be significantly different after Bonferroni-corrected p-value comparisons; p-values and percentages have been rounded off.

**Table 3:** Correlations between participant (patient) characteristics and monthly nurse visits (*n* = 21).

	Monthly nurse visits			
	Correlation coefficient	*p* value
Age	0.305	0.179		(i)
Years with PD	0.412	0.064	†	(i)
HY Stage	0.728	<0.001	*∗∗*	(ii)
No. of people in household	−0.387	0.083	†	(i)

^†^ *p* < 0.1, and ^*∗∗*^*p* < 0.01. (i) Pearson's correlation coefficient; (ii) Spearman's correlation coefficient; cc and *p* values have been rounded off.

## Data Availability

The data used to support the findings of this study are available from the corresponding author upon request.
